# Development of a Strategy for L-Lactic Acid Production by *Rhizopus oryzae* Using *Zizania latifolia* Waste and Cane Molasses as Carbon Sources

**DOI:** 10.3390/molecules28176234

**Published:** 2023-08-24

**Authors:** Feng-Wei Yin, Xiao-Long Sun, Wei-Long Zheng, Long-Fei Yin, Xi Luo, Ying-Ying Zhang, Yan-Fei Wang, Yong-Qian Fu

**Affiliations:** 1 Taizhou Key Laboratory of Biomass Functional Materials Development and Application, Taizhou University, Taizhou 318000, China; 2 Taizhou Institute of Product Quality and Safety Inspection, Taizhou 318000, China

**Keywords:** L-lactic acid, agro-industrial waste, *Zizania latifolia* waste, cane molasses, fermentation, *Rhizopus oryzae*

## Abstract

As a biodegradable and renewable material, polylactic acid is considered a major environmentally friendly alternative to petrochemical plastics. Microbial fermentation is the traditional method for lactic acid production, but it is still too expensive to compete with the petrochemical industry. Agro-industrial wastes are generated from the food and agricultural industries and agricultural practices. The utilization of agro-industrial wastes is an important way to reduce costs, save energy and achieve sustainable development. The present study aimed to develop a method for the valorization of *Zizania latifolia* waste and cane molasses as carbon sources for L-lactic acid fermentation using *Rhizopus oryzae* LA-UN-1. The results showed that xylose derived from the acid hydrolysis of *Z. latifolia* waste was beneficial for cell growth, while glucose from the acid hydrolysis of *Z. latifolia* waste and mixed sugars (glucose and fructose) from the acid hydrolysis of cane molasses were suitable for the accumulation of lactic acid. Thus, a three-stage carbon source utilization strategy was developed, which markedly improved lactic acid production and productivity, respectively reaching 129.47 g/L and 1.51 g/L·h after 86 h of fermentation. This work demonstrates that inexpensive *Z. latifolia* waste and cane molasses can be suitable carbon sources for lactic acid production, offering an efficient utilization strategy for agro-industrial wastes.

## 1. Introduction

The modern industrial civilization based on fossil resources, which rapidly developed in the 20th century, not only promoted the rapid growth of the world economy but also led to the rapid development of the plastic industry, resulting in increasing pollution of the oceans and terrestrial environment. Plastic decomposes very slowly, resulting in large amounts of waste accumulating in the oceans and on beaches, which poses great harm to birds, fish and other marine animals. Recently, microplastics and nanoplastics have become a global problem, posing a threat to human health [1]. Therefore, the transition from a fossil economy to a circular carbohydrate economy and the development of environmentally friendly biodegradable materials through sustainable methods is an inevitable trend for social and economic development.

Polylactic acid (PLA) is an ideal green polymeric material and is considered as one of the ideal biomaterials to replace traditional plastics because of its good biodegradability and other excellent use characteristics, such as biocompatibility, transparency, thermoplasticity and product safety [2,3]. As one of the most promising biodegradable materials, the production of PLA is receiving increasing attention around the world. However, the high production costs hinder the large-scale application of PLA due to the high price of lactic acid, the precursor material of PLA [4]. Currently, most of the lactic acid worldwide is produced through fermentation [3,5], and industrial lactic acid fermentation is primarily carried out using lactic acid bacteria [5,6]. Lactic acid has two optical isomers, L-lactic acid and D-lactic acid, whereby the former is widely used in the food and cosmetics industries due to its better biocompatibility with the human body. Unlike lactic acid bacteria, the fungus *Rhizopus oryzae* produces only L-lactic acid [7], in addition to a number of other unique advantages, such as high efficiency, safety, environmental protection, etc., and is increasingly being applied in the synthesis of L-lactic acid [8,9,10].

There are many factors that can affect the cost of producing lactic acid through fermentation, such as raw materials, fermentation reactors and lactic acid purification [5], among which carbon sources are among the most important operational expenses in the LA fermentation process due to the high cost of the raw materials [11]. Glucose derived from starch is the traditional carbon source for LA production [5,6,7], which not only accounts for a significant portion of the production cost of lactic acid but also competes with the food and feed supply [12]. These are no economically feasible production methods for large-scale LA fermentation. Recently, various low-cost renewable materials were chosen and applied to LA fermentation [4]. Lignocellulose represents the world’s largest reserve of renewable resources, including agricultural wastes and residues, industrial wastes, forestry processing wastes and grasses, as well as urban waste streams, which are highly abundant resources. After appropriate processing, lignocellulose can be converted into monosaccharides such as xylose and glucose, and the production of bulk chemicals such as organic acids from lignocellulose has enormous development potential [13].

*Zizania latifolia*, a wild member of the rice tribe Oryzeae, is an important crop and a popular aquatic vegetable in China, Japan, Korea and Southeast Asia that has been cultivated for more than 2000 years [14]. *Z. latifolia* is rich in nutrients and is widely planted in the southern region of the Yangtze River and Jiangsu and Zhejiang Provinces, with a total planting area of approximately 70,000 hm^2^ [15]. While *Z. latifolia* has enormous economic value, its wide cultivation also brings many negative issues. For example, for every 1.0 kg of saleable vegetables produced, 1.5–2.0 kg of agricultural waste is generated, resulting in over 5 million tons of *Z. latifolia* waste generated annually in China. During the abundant harvest season, tens of thousands of tons of *Z. latifolia* leaves are abandoned in piles by the roadside, in river channels and in fields, while leaf sheaths are left in the fields to rot or are burned. This not only affects the healthy development of the *Z. latifolia* industry but also brings serious environmental pollution problems [16]. However, due to limitations in technology, costs and application fields, the waste utilization of *Z. latifolia* has not been widely promoted. Nevertheless, the waste of *Z. latifolia* has been used as a biological adsorbent [16], animal feed [17] and raw material for activated carbon [18]. It is reported that *Z. latifolia* waste contains rich nutritional components, as well as cellulose, hemicellulose, lignin and other typical lignocellulosic constituents [19], which can be transformed into mixed sugar solutions such as glucose and xylose after physical, chemical or biological treatment and can be used for the fermentation of ethanol, organic acids and other bulk chemicals. Therefore, *Z. latifolia* waste may be developed as a new type of cheap carbon source for LA production.

Molasses are the most abundant by-product of sugar mills, with the annual output reaching 400 million tons in China [20]. Traditionally, the waste molasses are simply discharged or applied to low-value feed production, aggravating environmental pollution and the waste of resources [21]. However, molasses are becoming a major carbon choice for the industrial fermentation of high-valued products due to their sugar composition and economic value compared to other pure sugars [21,22,23]. Recently, high-grade molasses were successfully applied in LA fermentation by *R. oryzae* [24,25]. 

At present, the synthesis of lactic acid by *R. oryzae* is difficult to industrialize due to its low efficiency and high cost. In order to increase the production of lactic acid by *Rhizopus oryzae*, several studies have been devoted to the screening of high-yield strains [3,26], the control of the cell morphology [27], immobilized cells [28] and the study of new bioreactors [10]. However, in the vast majority of studies using *R. oryzae* fermentation to synthesize L-lactic acid, the yield and production intensity of lactic acid are far lower than the production efficiency of lactic acid bacteria [5,6]. As a consequence, *R. oryzae* has not yet been used in the industrial production of L-lactic acid. The control of the fermentation costs remains the key to achieving the industrial production of L-lactic acid derived from *R. oryzae*, especially the raw material cost [4]. 

The aim of this work was to develop a new economic production process for the synthesis of a high-purity polylactic acid monomer using *R. oryzae*, focusing on *Z. latifolia* waste and cane molasses as carbon sources. Furthermore, in order to improve the efficiency of L-lactic acid production from these two cheap carbon sources, an efficient multi-stage carbon source utilization strategy was developed. This work brings us closer to economical industrial lactic acid production from *Z. latifolia* waste and cane molasses, furthering the valorization of agro-industrial wastes.

## 2. Results and Discussion

### 2.1. Effects of Different Carbon Sources on Biomass and LA Production

**Figure 1:**
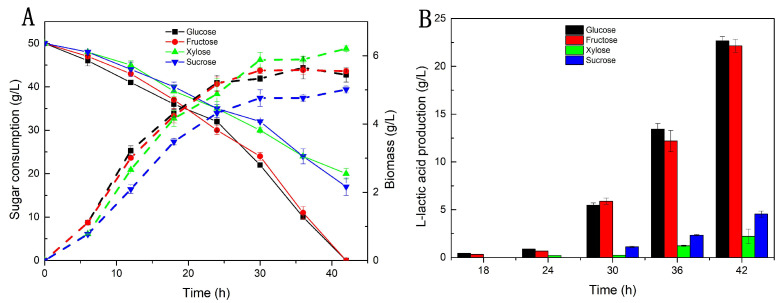
The effects of different sugars on cell growth (**A**) and L-lactic acid production (**B**) of *R. oryzae*.

The aim of this study was to realize the sustainable valorization of two waste by-products, *Z. latifolia* waste and cane molasses, as carbon sources for LA production by *R. oryzae*. While the main carbon sources found in pretreated *Z. latifolia* waste are xylose and glucose, the main constituents of pretreated cane molasses are glucose and fructose. Therefore, we first assessed the effects of glucose, fructose, sucrose (Sinopharm Chemical Reagent Co., Ltd., Shanghai, China) and xylose (Shandong Futian Pharmaceutical Co., Ltd., Dezhou, China) on cell growth and LA production. The sugar consumption and biomass accumulation on different carbon sources are shown in Figure 1. The effects of different carbon sources on the growth of *R. oryzae* varied significantly. As the most common hexose, glucose can be quickly utilized by *R. oryzae* cells [7], and the cultures entered the rapid consumption period of glucose after 24 h (Figure 1A), at which point they also began to rapidly synthesize LA (Figure 1B). The same tend was observed when fructose was used as the sole carbon source. Compared with glucose, xylose consumption was significantly delayed, and there was still 20 g/L residual xylose in the culture broth at the end of the fermentation (42 h). This may be related to the different cellular sugar transport mechanisms [29]. Interestingly, when taking the cell growth into account, we found that the highest finial biomass of 6.21 g/L was obtained on xylose, with a biomass/sugar conversion rate of 0.21, which was higher than that of glucose and fructose (0.11), suggesting that xylose is more conducive to the biomass accumulation of this strain. A similar result was obtained in a previous study, where xylose preferentially enhanced the cell growth of *Bacillus coagulans* IPE22 [30] and *Rhizopus oryzae* CBS 127.08 [31]. This difference may be caused by changes in the synthesis of biological macromolecules such as carbohydrates and proteins when the cells are grown on xylose [32]. However, xylose was not conducive to the synthesis of LA (Figure 1B). This was also in agreement with the literature [33] and may be due to the negative impact xylose on lactate production [34] or the preferential respiratory dissimilation of xylose instead of LA production [31]. Sucrose is a disaccharide that needs to be degraded into monosaccharides, but it can also be used as a carbon source for LA fermentation [10]. Nevertheless, it could not be effectively utilized by *R. oryzae* in this study. Thus, the results indicated that xylose was a superior carbon source for cell growth, while glucose and fructose are suitable carbon sources for LA production in *R. oryzae*.

### 2.2. Z. latifolia Waste as Carbon Source for LA Fermentation

#### 2.2.1. Effect of *Z. latifolia* Waste Acid Hydrolysate on Cell Growth

**Figure 2:**
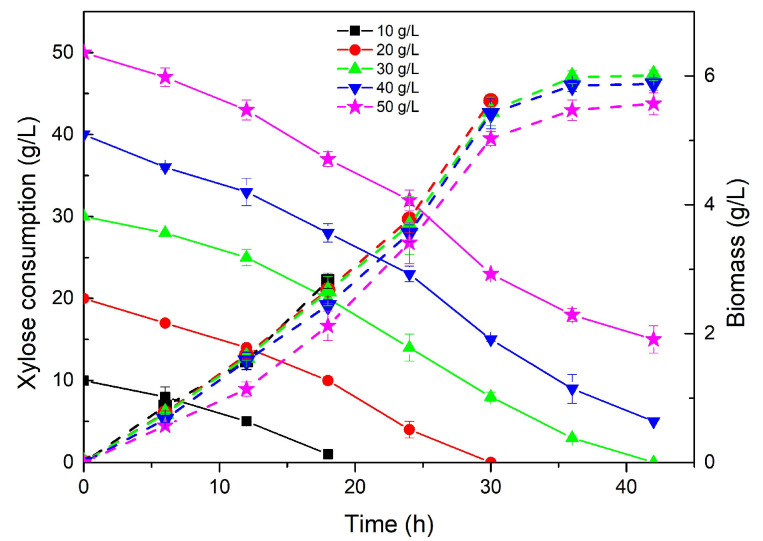
The effect of different concentrations of xylose from the acid hydrolysate of *Zizania latifolia* waste on the growth of *R. oryzae*.

Based on the above experimental results, it was feasible to use xylose from hydrolyzed *Z. latifolia* waste for *R. oryzae* cell growth in the seed culture. The effect of different initial concentrations of xylose on the growth of *R. oryzae* was investigated, as shown in Figure 2. The xylose consumption from the hydrolysate showed a similar tendency to that of pure xylose. The strain showed a similar growth rate at different concentrations of xylose-containing hydrolysate in the first 30 h, and the growth slowed down after 30 h, which might have been caused by the depletion of the nitrogen sources [7,35,36]. The final biomass increased from 2.82 g/L with 10 g/L xylose to 6.01 g/L with 30 g/L xylose, indicating that the xylose from the *Z. latifolia* waste hydrolysate was suitable for this strain. However, a higher xylose concentration did not result in higher biomass accumulation, even after prolonged culture, so that the final biomass reached 5.57 g/L at 42 h with 50 g/L xylose, similar to the result obtained with 20 g/L xylose at 30 h, while the biomass/sugar ratio was reduced by 43% (from 0.28 to 0.16). This might be due to the harmful by-products in the hydrolysis process of lignocellulose, such as furfural, acetic acid, formic acid and phenolic compounds [37]. Accordingly, as the concentration of the xylose-containing hydrolysate increased, the inhibitory effects become more pronounced (Figure 2). However, considering its affordability and adaptability, the xylose-containing hydrolysate could still be a viable carbon source for biomass accumulation during the first phase of culture.

#### 2.2.2. LA Fermentation Using Seed Cultures Grown Using Xylose from *Z. latifolia* Waste Acid Hydrolysate

In order to confirm the optimal concentration of the xylose-containing hydrolysate for LA fermentation, a further experiment was conducted with seed cultures grown with different amounts of xylose in combination with 50 g/L of pure glucose (Table 1).

It is well known that cell density plays a key role in the fermentation of biological products [38], and the biomass accumulated in the early stage plays an important role for later LA synthesis [7]. This is the reason for the lowest LA production of 18.25 g/L obtained using the seed culture grown with 10 g/L xylose, which also required a longer fermentation time. The highest LA production was obtained using seed cultures grown with 20 and 30 g/L xylose. However, a higher concentration of xylose (40–50 g/L) could not increase LA production and only resulted in a similar biomass to that obtained with 20 g/L xylose. This might be related to the vitality of the seed cultures, since highly active cells are more conducive to the generation and accumulation of target products [21]. In this study, although hydrolysates containing 20 g/L xylose resulted in 5.62 g/L biomass, less than with 30 g/L xylose, this seed culture with 20 g/L xylose possessed higher activity and the resulting fermentation required less time, which was more conducive to improving the production efficiency of LA fermentation, with the highest LA production of 23.05 g/L obtained in the LA fermentation process. Thus, the hydrolysate containing 20 g/L xylose was utilized for the seed culture in the following experiments.

#### 2.2.3. Effect of Enzymatic *Z. latifolia* Waste Hydrolysate on LA Fermentation

**Figure 3:**
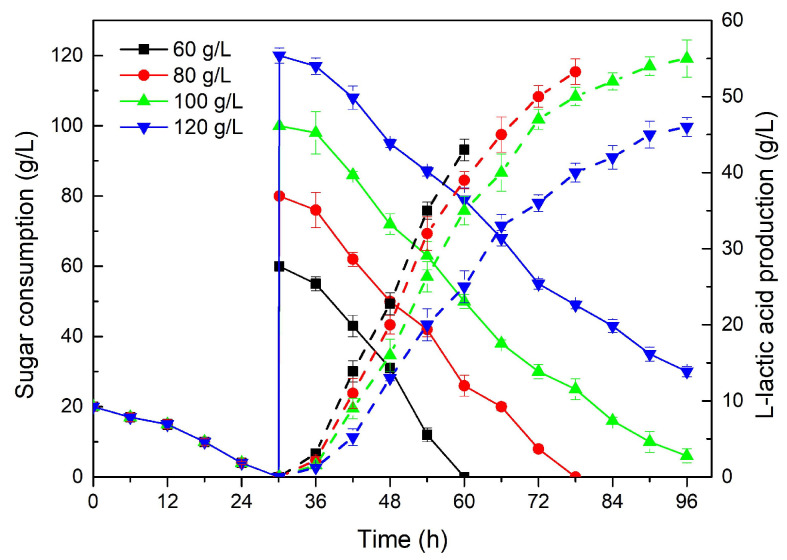
The effect of different concentrations of glucose from the enzymatic hydrolysate of *Zizania latifolia* on L-lactic acid fermentation.

Based on the results obtained above, there was potential to use a two-stage carbon source feeding strategy for LA fermentation. Thus, in stage 1, biomass accumulation was conducted with 20 g/L xylose, while, in stage 2, LA synthesis was promoted by supplementing glucose in the same shake flask. To further test the feasibility of this strategy and the applicability of the enzymatically produced glucose-containing hydrolysate, LA fermentations were conducted with 60–120 g/L of the glucose-containing hydrolysate (Figure 3). In each group, 20 g/L of xylose was applied as the carbon source for biomass accumulation, and when the xylose was exhausted (30 h), the carbon source was changed to glucose for LA fermentation. As shown in Figure 3, different from the rapid consumption of pure glucose, the consumption of glucose from the enzymatic hydrolysate was delayed. This was not surprising, as some products of incomplete enzymatic hydrolysis, such as cellobiose and oligosaccharides, were present in the sugar solution after enzymolysis [39] and were degraded into glucose by *R. oryzae* during LA fermentation. As can be clearly seen in Figure 3, when cells entered the acid production stage as the nitrogen source was exhausted, the glucose was rapidly consumed, similar to a previous report [40]. The final fermentation times were different due to the different glucose utilization rates. With a prolonged fermentation time, the yield of LA was increased from 42.12 g/L with 60 g/L glucose to 55.14 g/L with 100 g/L (Table 2). However, it should be noted that a higher glucose concentration (100–120 g/L) resulted in a decrease in LA production efficiency in the later stage of the fermentation, with the productivity being 0.57 and 0.48 g/L·h, respectively, after 96 h of fermentation, which was different from our previous study with pure glucose as a carbon source [7]. This discrepancy might be due to the harmful by-products generated during the pretreatment process of *Z. latifolia* waste [37], resulting in a reduction in the LA yield. The highest LA productivity and yield of 0.70 g/L·h and 0.70 g/g were obtained in the 60 g/L group, but its LA titer (42.12 g/L) was lower than in the 80 g/L group (53.36 g/L) due to the insufficient substrate carbon source. Moreover, the group with 80 g/L had similar LA productivity and yields of 0.68 g/L·h and 0.67 g/g when compared with the 60 g/L group (Table 2). Therefore, it was suggested that 80 g/L glucose from the enzymatic *Z. latifolia* hydrolysate had little effect on LA production and could be a suitable concentration for LA fermentation.

### 2.3. Waste Cane Molasses as Carbon Source for LA Fermentation

Glucose from the enzymatic *Z. latifolia* waste hydrolysate could not increase the LA yield as the sole carbon source, since the inhibitory effect occurred when the glucose concentration exceeded 80 g/L. Therefore, another agro-industrial waste, cane molasses, was tested as a carbon source for LA fermentation since the main sugar of this waste is sucrose, which can be hydrolyzed into glucose and fructose, both of which were shown to be suitable for LA production in this study.

We first analyzed the effects of pretreated and unpretreated molasses on LA fermentation, using a total sugar concentration of 50 g/L, and the biomass accumulation in the early stage using the same method as above. As shown in Table 3, the different treatments applied to the molasses resulted in significant differences in LA fermentation. Although untreated molasses could be used as a carbon source for LA production, the result was not ideal, and, after 62 h fermentation time, 28.21 g/L LA was obtained, which was 22% less than with treated molasses. Interestingly, the biomass and LA production were higher than with pure sucrose, likely due to the protein content of molasses [41]. However, untreated molasses also contain some colloidal matter, ash, metal ions and other suspended substances that could have harmful effects on the cells [42]. Therefore, molasses are often added at a relatively low and suitable concentration [41,43] or pretreated before use [21,44]. Thus, in order to reduce the toxic effects, treated molasses were utilized as the fermentation carbon source. 

**Figure 4:**
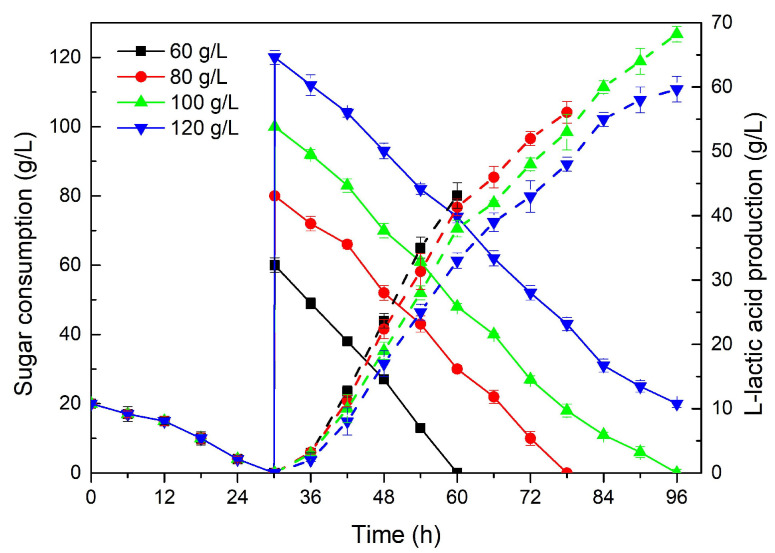
The effect of different concentrations of total sugar from the acid hydrolysate of cane molasses on L-lactic acid fermentation.

To further test the applicability of treated cane molasses, 60–120 g/L of total sugar from cane molasses was added for LA fermentation (Figure 4). The sugar consumption was fast during the whole fermentation process, indicating that treated cane molasses are an optimal substrate for LA synthesis by *R. oryzae*. The accumulation of LA was in line with the rapid consumption of the substrates, since glucose and fructose were suitable carbon sources for LA synthesis. The final LA titer was increased from 43.15 g/L with 60 g/L total sugar to 68.24 g/L with 100 g/L total sugar (Table 4). However, a higher total sugar concentration (120 g/L) resulted in a significant decrease in the LA yield (0.60 g/g), with lower productivity of 0.62 g/L·h after 96 h fermentation, which may be attributed to the harmful components of molasses, since the pretreatment of the cane molasses could not completely remove all undesirable substances and further processing would increase the cost [21,43]. Nevertheless, an LA yield of 0.68 g/g and productivity of 0.71 g/L·h were obtained when using 100 g/L total sugar from treated cane molasses, similar to the results obtained from low-concentration sugar conditions (60–80 g/L) (Table 4), indicating that this substrate level could not affect the synthesis of LA by *R. oryzae* and it could be applied as a carbon source for LA fermentation.

### 2.4. Multi-Stage Carbon Source Feeding Strategy

**Figure 5:**
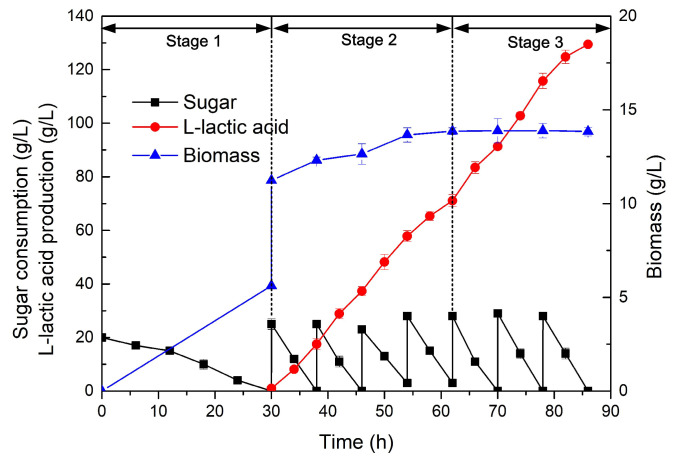
Three-stage fermentation profile of L-lactic acid production by *R. oryzae* using different sugars from *Zizania latifolia* waste and cane molasses.

A biomass with a high cell density and activity is a key factor in achieving ideal fermentation results, while the substrate consumption is directly related to the generation of target products. In this work, growing the seed biomass on xylose was beneficial for the synthesis of LA, while 80 g/L of glucose from the enzymatic *Z. latifolia* waste hydrolysate and 100 g/L total sugar from the acid hydrolysate of cane molasses were the respective upper limits with no negative effect on LA fermentation. Based on these results, and hoping to achieve efficient LA production from agro-industrial wastes, this study innovatively developed a three-stage fermentation strategy. In stage 1, the biomass was grown using xylose, while, in stage 2, LA synthesis was promoted using the acid hydrolysate of cane molasses, whereby the nutrients found in molasses were also beneficial for further biomass accumulation. Finally, in stage 3, further LA synthesis was conducted using glucose from the enzymatic hydrolysate. In order to improve the consumption rate of the carbon sources in the LA synthesis stage, the cell density was doubled by combining two identical seed cultures and reducing the fermentation volume by 50% after 30 h, as described in previous studies [7,45]. The results are shown in Figure 5.

In the beginning, the initial total sugar value with xylose as a carbon source was 20 g/L for the seed culture; after 30 h of biomass accumulation with xylose, when the nitrogen source was exhausted, the carbon source was changed to cane molasses. During the entire LA fermentation process, the concentration of the carbon source was kept at 0–30 g/L to reduce the toxic effects of impurities and increase the rate of substrate consumption [21,46]. Although the growth rate of cells slowed down due to the depletion of the nitrogen source in the broth [45], there was a further increase caused by the additional nitrogenous nutrients found in cane molasses [41]. The accumulation of the biomass after doubling in stage 1 promoted the rapid accumulation of LA; this indicated that a large number of highly active cells cultured with xylose in the seed culture stages could quickly switch to using sugars (glucose and fructose) from treated molasses. It was proven that a high cell density played an important role in increasing the production of biological products [47], and, for LA production by *Rhizopus oryzae*, the LA concentration is directly related to the cell dry weight [7]. However, the growth of the biomass is not synchronized with the accumulation of lactic acid [46]. In addition, after the nitrogen source is depleted, the biomass tends to reach a relatively constant level (Figure 5), and cells shift their metabolic pathways from cell growth to LA accumulation [41]. The acid accumulation stage was only 86 h.

The first stage used xylose as the carbon source to obtain a high-quality biomass with high activity and sufficient cells, and, during stage 2 from 30 to 62 h, 73.14 g/L of LA was produced from cane molasses, although harmful substances from the cane molasses began to accumulate in the fermentation broth, but this amount was not sufficient to affect cell activity. Similarly, in stage 3 from 62 h to the end, 56.33 g/L of LA was produced from *Z. latifolia* waste, indicating that cells cultured under low-concentration cane molasses conditions using fed-batch fermentation could still maintain high vitality, allowing cells to continue to synthesize LA after changing their carbon sources. After 86 h of fermentation, 180 g/L of total sugar (80 g/L from *Z. latifolia* waste and 100 g/L from cane molasses) yielded 129.47 g/L of LA. When comparing the results of this study with what was reported in previous studies using agro-industrial wastes (Table 5), it is clear that this work achieved a higher yield of LA with higher productivity. Therefore, *Z. latifolia* waste and cane molasses were successfully and efficiently utilized for LA production by *R. oryzae* via this novel fermentation strategy. 

### 2.5. Cost Analysis of Carbon Sources

The calculation of the cost of using *Zizania latifolia* waste and cane molasses for L-lactic acid production is shown in Table 6 and compared with pure glucose. The prices of glucose (Xiwang group, Zouping, China) and cane molasses (Guangdong Huatang Industrial Co., Ltd., Jiangmen, China) are approximately USD 550 and USD 140/t, respectively. The *Zizania latifolia* waste (Taizhou, China) is free of charge. According to our previous study using glucose as a carbon source [7], for 1 m^3^ of fermentation broth in traditional fermentation using pure glucose, 220 kg glucose is consumed, resulting in 160 kg L-lactic acid. By contrast, the novel fermentation strategy using sugars form *Zizania latifolia* waste and cane molasses consumes 0 kg pure glucose, 298 kg *Z. latifolia* waste and 212 kg cane molasses and results in 129.47 kg L-lactic acid. Therefore, the carbon sources costs for 1 t L-lactic acid are calculated at USD 756.25 for the traditional fermentation using pure sugar and USD 229.24 for the new strategy using agro-industrial wastes. The results thus indicate that the cost of L-lactic production is reduced by 69.69% when *Z. latifolia* waste and cane molasses are used as carbon sources in conjunction with the optimized fermentation protocol.

## 3. Materials and Methods

### 3.1. Microorganism

The L-lactic acid production strain was *Rhizopus oryzae* LA-UN-1, which was also used in our previous study [7] and was domesticated using a method developed by Yin et al. [54]. The domesticated strain was preserved in the Taizhou Key Laboratory of Biomass Functional Materials Development and Application.

### 3.2. Fermentation

#### 3.2.1. Medium

For the seed culture, the medium included (g/L) sugar 10–50; peptone 4.0; KH_2_PO_4_ 0.2; MgSO_4_·7H_2_O 0.2; unadjusted pH. For batch and fed-batch fermentation, the medium was the same as the seed medium, while, during the fed-batch fermentation process, CaCO_3_ was added to maintain the fermentation broth at pH 6.5–7.0, and the feeding carbon sources were added to maintain the total sugar concentration of 0–30 g/L.

#### 3.2.2. Fermentation Protocol

Preparation of the spore suspension: *R. oryzae* was cultured on potato dextrose agar (PDA) slants for 6–7 days. After maturation, the spores were washed off from the slant medium with sterile water. After residual mycelium was removed by filtration through sterile cotton wool, the spore suspension was adjusted to 10^7^–10^8^/mL and stored in a refrigerator at 4 °C.

Seed culture: 1 mL of the spore suspension was used to inoculate a 250 mL Erlenmeyer flask containing 50 mL of fermentation medium, and the seed culture was incubated at 200 rpm and 30 °C.

Fermentation culture: The seed broth was transferred into a 500 mL Erlenmeyer flask containing 100 mL of fermentation medium, and the fermentation culture was incubated at 250 rpm and 30 °C.

### 3.3. Carbon Sources and Pretreatment

#### 3.3.1. *Zizania latifolia* Waste

*Zizania latifolia* was obtained from Huangyan District, Taizhou City, Zhejiang Province. The *Z. latifolia* waste encompassed leaf sheaths, leaves and other non-edible parts of the plant. The pretreatment of *Z. latifolia* waste was as follows.

After drying, the *Z. latifolia* waste was firstly ground to a particle size of 10–20 mm and then extracted with 90% ethanol (1:10, *v*/*w*) at 80 °C for 2 h to obtain solids. The above solids were soaked in formic acid (1:10, *v*/*w*) at 70 °C for 2 h, after which the mixture was filtered to obtain solids and liquids. The obtained solid material could be used as a material with cellulose as the main component. The formic acid was recovered by vacuum distillation from the liquid fraction, leaving water-insoluble components, mainly lignin, and water-soluble components. Subsequently, water was added to dissolve the hemicellulose hydrolysate, with xylose as the main component.

The above cellulosic material was washed with water, and the enzymatic digestion conditions were based on a method by Ling et al. [55], as follows. The reaction conditions were 50 °C with a pH of 4.8 (50 mM acetic acid–sodium acetate buffer) for 72 h, and the enzyme dosage was 60 FPU/g dry cellulosic material, resulting in the cellulose-hydrolyzed sugar, glucose.

#### 3.3.2. Cane Molasses

The cane molasses were purchased from Guangdong Huatang Industrial Co., Ltd. (Guangdong, China). The waste cane molasses were subjected to acidolysis with 5 M sulfuric acid, and the pH was adjusted to 3.5, followed by heating at 80 °C under ventilated conditions for 12 h. Then, the acid-treated cane molasses were adjusted to pH 5.5 with lime milk and centrifuged at 5000× *g* for 10 min, and the supernatant fraction was collected as the carbon source.

### 3.4. Analytical Methods

Sugar consumption and L-lactic acid synthesis were analyzed by HPLC (LC-20AT, Shimadzu, Kyoto, Japan) with a Refractive Index Detector (RID-20A, Shimadzu, Kyoto, Japan), as described in our previous study [7]. The column used in this study was an Aminex HPX-87 H Ion Exclusion Column (300 mm × 7.8 mm, Bio-Rad, Hercules, CA, USA). The column temperature was set at 60 °C using 5 mM H_2_SO_4_ as the mobile phase with a flow rate of 0.8 mL/min. The injection volume was 20 μL.

The biomass was expressed as cell dry weight (CDW), which was determined by weighing the mycelial mass after drying to a constant weight at 60 °C.

## 4. Conclusions

In this work, inexpensive carbon sources from *Z. latifolia* waste and cane molasses were applied in L-lactic acid fermentation by *R. oryzae* LA-UN-1. A novel fermentation strategy was developed with three-stage carbon source feeding to increase the biomass accumulation and carbon utilization. Using this strategy, we achieved an L-lactic acid titer of 129.47 g/L, with a yield of 0.72 g/g and productivity of 1.51 g/L·h after 86 h of fermentation. The results of this study provide a theoretical basis for large-scale L-lactic acid production using *R. oryzae*, whereby the novel strategy could also be applied to other agro-industrial wastes.

## Figures and Tables

**Table 1:** The effect of seed cultures grown with xylose from the acid hydrolysate of *Zizania latifolia* waste on L-lactic acid fermentation.

Seed Culture			L-Lactic Acid Fermentation	
Xylose Concentration (g/L)	Culture Time (h)	Biomass (g/L)	Fermentation Time (h)	L-Lactic Acid Production (g/L)
10	18	2.28	48	18.25
20	30	5.62	42	23.05
30	42	6.01	42	22.78
40	42	5.88	42	20.33
50	42	5.57	42	20.12

**Table 2:** Comparison of the parameters of L-lactic acid production by *R. oryzae* using different concentrations of glucose from the enzymatic hydrolysate of *Zizania latifolia*.

Glucose Concentration (g/L)	Glucose Consumption (g/L)	Fermentation Time (h)	L-Lactic Acid Production (g/L)	Productivity (g/L·h)	Yield (g/g)
60	60	60	42.12	0.70	0.70
80	80	78	53.36	0.68	0.67
100	94	96	55.14	0.57	0.58
120	90	96	46.47	0.48	0.52

**Table 3:** Comparison of the parameters of L-lactic acid production by *R. oryzae* using treated and untreated molasses as the carbon source.

Carbon Source	Sugar Consumption (g/L)	Biomass (g/L)	Fermentation Time (h)	L-Lactic Acid Production (g/L)	Productivity (g/L·h)	Yield (g/g)
Untreated molasses	40	5.11	54	25.21	0.46	0.50
Treated molasses	50	5.89	54	36.76	0.68	0.74

**Table 4:** Comparison of the parameters of L-lactic acid production by *R. oryzae* using different concentrations of sugar from the acid hydrolysate of cane molasses.

Sugar Concentration (g/L)	Sugar Consumption (g/L)	Fermentation Time (h)	L-Lactic Acid Production (g/L)	Productivity (g/L·h)	Yield (g/g)
60	60	60	43.15	0.72	0.72
80	80	78	56.07	0.72	0.70
100	100	96	68.24	0.71	0.68
120	100	96	59.66	0.62	0.60

**Table 5:** A comparison of the production of lactic acid using agro-industrial wastes in this study with results reported in the literature.

Strain	Substrate	Concentration (g/L)	Yield (g/g)	Productivity (g/L·h)	References
*R. arrhizus*	Waste potato starch	103.8	-	2.16	[48]
*R. arrhizus*	Syrup of carrot discards	22.18	0.79	0.31	[49]
*R. oryzae* UMIP 4.77	Wheat straw	10	0.26	0.27	[34]
*R. oryzae*	Tobacco waste water extract and glucose	173.5	0.86	1.45	[50]
*R. oryzae*	Cassava pulp hydrolysates	75.28	0.5	1.05	[36]
*R. oryzae* 3.819	Sophora flavescens residues	46.78	-	0.97	[40]
*R. oryzae* MTCC5384	Industrial waste paper sludge	27	-	-	[51]
*R. orysae*	Yam peel hydrolysate	64.02	0.80	-	[52]
*R. oryzae* NLX-M-1	Xylo-oligosaccharides Manufacturing waste residue	60.3	0.6	1.0	[53]
*R. oryzae* LA-UN-1	*Zizania latifolia* waste and cane molasses	129.47	0.72	1.51	This study

**Table 6:** Cost calculation of conventional fermentation using glucose and the novel fermentation with *Zizania latifolia* waste and cane molasses.

	Pure Glucose	*Z. latifolia* Waste	Cane Molasses	Total Cost $	L-Lactic Acid kg	L-Lactic Acid Cost $/t
Price ($/t)	550	0	140			
Traditional fermentation (kg)	220	0	0	121	160	756.25
Novel fermentation (kg)	0	298	212	29.68	129.47	229.24
Cost saving (%)	69.69					

## Data Availability

Not applicable.
